# The cross-cultural adaptation of Chinese international students: an empirical study on sequential-mediated effects

**DOI:** 10.3389/fpsyg.2024.1386044

**Published:** 2024-06-18

**Authors:** Chenglong Miao, Shuai Zhang

**Affiliations:** ^1^Department of Leisure Sports, Kangwon National University, Samcheok-si, Gangwon-do, Republic of Korea; ^2^Sports Psychology Techniques and Training Research Institute, Kangwon National University, Samcheok-si, Gangwon-do, Republic of Korea

**Keywords:** cross-cultural adaptation, positive coping, negative coping, sports participation, in-group identification, out-group bias

## Abstract

Using convenience sampling and snowball sampling methods, data from 432 Chinese international students in 10 countries, including the United Kingdom, South Korea, and the United States, were collected to construct a multivariate sequential-mediated mixed model for cross-cultural adaptation. SPSS 23.0 and AMOS 23.0 were employed for aggregated validity, discriminant validity, and sequential-mediated effects analysis. The study found that: Cultural adaptation stress is negatively correlated with positive coping and positively correlated with negative coping, with negative coping having a significant negative impact during the cross-cultural adaptation process. Positive coping is positively correlated with sports participation, while negative coping is negatively correlated with sports participation. Sports participation is positively associated with in-group identification and negatively associated with out-group bias. In-group identification has a positive impact on cross-cultural adaptation, whereas out-group bias cannot effectively predict cross-cultural adaptation.

## 1 Introduction

According to the latest statistics from the Chinese Ministry of Education, from 1978 to 2019, nearly 6.56 million students have studied abroad. International students are more prone to experiencing psychological issues such as anxiety, depression, paranoia, fear, and interpersonal relationship difficulties (Ismail and Yang, 2003). In addition to academic pressure, international students face significant psychological stress due to the challenges of interpersonal communication and adjusting to different social and cultural norms (Mori, 2000). Although Berry proposed four cultural coping strategies: marginalization, separation, assimilation, and integration (Berry, 1997), however, When Chinese culture comes into contact with the culture of the host country, international students often struggle with adapting to the social norms and cultural customs of the new culture, leading to psychological stress in the process of cross-cultural adaptation, known as cultural adjustment pressure (Lin and Yi, 1997).

Although there has been some accumulation of research on the psychological well-being of international students, there are still many research gaps within the Chinese international student population, especially among those facing challenges in cross-cultural adaptation. Particularly, further exploration is needed regarding how they cope with these psychological pressures. Hence, the urgency of studying the psychological pressures faced by Chinese international students during the process of cross-cultural adaptation, as well as their coping strategies, is highlighted.

Cross-cultural adaptation is an ongoing process of interaction between individuals from different cultural backgrounds, specifically evident in the physical and psychological adjustment experienced by Chinese international students during their familiarization with the host country’s culture (Ma, 2017). Throughout the process of physical and mental adaptation, international students often face a multitude of cross-cultural pressures, primarily concentrated on social stressors and psychological challenges, including interpersonal tension and anxiety (Lai et al., 2023). It is particularly emphasized that major sources of stress for international students in their academic life also include language adaptation pressure (Wan, 1999). The ways in which international students cope with these pressures can be explained through stress coping theories, with the most widely recognized coping strategies currently being positive coping methods and negative coping methods (Sapranavicute et al., 2012).

Chinese international students are more prone to experiencing psychological issues such as anxiety, depression, paranoia, fear, and interpersonal relationship difficulties (Sam and Eide, 1991). In addition to facing academic pressure, the adjustment to different social and cultural norms becomes a primary source of psychological stress for Chinese international students (Cao et al., 2021). Moderate stress is considered beneficial, serving as a driving force, but excessive stress can act as a hindrance (Bai, 2016). As carriers of their homeland culture, Chinese international students living in a foreign country experience direct exposure to a different culture, leading to inevitable “collisions and friction” between cultures (Cao and Zhang, 2012). Consequently, they often struggle to adapt to new social norms and cultural customs (Ward and Kennedy, 1993), giving rise to psychological burdens during the process of cross-cultural adaptation, known as cultural adjustment pressure (Ho et al., 2007).

Sports participation, as one of the positive coping mechanisms for stress management, plays a crucial role in promoting the physical and mental well-being of individuals. Kita (2014) emphasizes that engagement in sports can enhance the flexibility and functioning of the cerebral cortex, improving cognitive abilities such as attention, memory, reaction time, thinking, and imagination. Regular participation in sports not only contributes to heightened cognitive abilities but also results in emotional stability, a cheerful personality, and reduced fatigue. Research by Bilotserkovets et al. (2020) on Chinese international students in Ukraine indicates that the success of cross-cultural adaptation relies on effective in-group identification. In the context of in-group identification during sports participation among Chinese international students, it facilitates mutual communication and understanding with the local cultural group, enhancing group trust, and reducing out-group bias. Research and theoretical development in the field of cross-cultural psychology are extensive and sophisticated, exemplified by Berry’s psychological acculturation model and Denoux’s psychological cross-cultural adaptation model. However, this paper focuses on stress coping during the cross-cultural adaptation process. Therefore, it centers on Lazarus and Folkman’s stress and coping theory, exploring the chain mediation effects through four mediating pathways: active coping, passive coping, sports participation, in-group identification, and out-group prejudice.

## 2 Literature review and research hypotheses

### 2.1 Cultural adaptation stress and positive coping, negative coping

Coping strategies refer to the thoughts or behaviors that individuals employ to address the internal and external demands perceived as stressful situations or as attempts to control, reduce, or endure the challenges, harm, or threats associated with perceived stressors (Lazarus and Folkman, 1984). When individuals become aware of facing particularly difficult issues, the perception of challenges or obstacles as stressors is characterized by a heightened state of tension manifested through various psychological and physiological reactions (Biggs et al., 2017). When confronted with similar stressors, individuals may exhibit either positive or negative coping mechanisms based on differences in their levels of psychological adaptation. Positive coping involves individuals addressing problems, managing stress, or overcoming difficulties through proactive and effective approaches (Folkman et al., 1986). On the other hand, negative coping refers to individuals adopting harmful methods, either physically or psychologically, to deal with problems or confront challenges (Peterson et al., 1993). Lazarus and Folkman’s Stress and Coping Theory proposes that coping strategies can be categorized into problem-focused and emotion-focused types, which can be either positive or negative. Therefore, based on this theoretical foundation, this study simplifies coping strategies into positive and negative coping.

A substantial body of research confirms that in stressful situations, different coping mechanisms often result in diverse individual experiences (Folkman and Lazarus, 1988). For international students who need to adapt to a new culture and integrate into campus life, positive coping has been shown to effectively alleviate various issues encountered during the cultural adjustment process, reducing individual cross-cultural adaptation pressure and promoting psychological well-being (Wang et al., 2022). Conversely, negative coping tends to increase cultural resistance in individuals, thereby diminishing life satisfaction and potentially having a significant impact on mental health (D'haese et al., 2016).

In summary, during the cross-cultural contact process, when international students encounter cross-cultural adaptation pressure, they are inclined to adopt positive coping strategies if they believe they can overcome the stress. Conversely, if they perceive challenges in overcoming the pressure, they tend to lean toward negative coping, thereby generating a negative impact.

*Hypothesis H1a:* Cultural adaptation stress negatively influences positive coping among Chinese international students.

*Hypothesis H1b:* Cultural adaptation stress positively influences negative coping among Chinese international students.

### 2.2 Positive coping, negative coping and sports participation

Song and Lee (2021) and Petruzzello et al. (1991) have demonstrated that college students who approach things optimistically frequently engage in sports activities. When college students adopt positive coping, it tends to induce more positive psychological experiences. In related research on physical exercise interventions, Hayes et al. (2015) also pointed out that cognitive behaviors such as self-efficacy, subjective cognitive control, and attitudes are positively correlated with the level of physical activity. In other words, when college students are in a positive psychological state, characterized by high levels of self-efficacy, proactive cognitive control, and a positive attitude, it promotes a positive understanding of the benefits of sports participation and encourages active engagement.

The research conducted by Carpenter et al. (2011) suggests a significant positive correlation between negative coping and pressure in the context of sports. In other words, the more negatively college students cope with stress or generate negative cognitions in stressful situations, the greater the pressure related to sports becomes. With increased pressure, the coping response becomes more passive, consequently reducing college students’ enthusiasm for physical activities and potentially leading to the cessation of sports participation (Moen et al., 2019).

In summary, international students, when facing stress and pressure, tend to actively seek solutions, such as engaging in sports interventions. In the process of sports participation, they release stress and alleviate discomfort. However, for international students with lower levels of mental health, under stress and pressure, they are more prone to being dominated by emotions and adopting negative coping strategies, hindering the development of their mental well-being and leading to the emergence of social aversion symptoms (self-isolation, escaping reality). In other words, the higher the degree of negative coping, the less likely the involvement in sports participation.

*Hypothesis H2a:* Positive coping strategies among Chinese international students positively influence sports participation.

*Hypothesis H2b:* Negative coping strategies among Chinese international students negatively influence sports participation.

### 2.3 Sports participation and in-group identification, out-group bias

Research on group identification and group bias indicates that in-group refers to individuals recognizing themselves as belonging to a specific social group while also acknowledging the emotional and value significance that being a group member brings to them (Tajfel, 1978). Merely perceiving oneself as being categorized into a certain group leads to individuals allocating more resources to their own group and providing more positive evaluations for their group (Ostrom et al., 1993). At this point, individuals engage in self-categorization, also known as in-group identification. Conversely, there is an out-group bias (Castano et al., 2002).

Sports participation plays a crucial role in the physical and mental well-being of individuals (Khan et al., 2022). Engaging in sports not only stimulates individual activity but also enhances brain responsiveness and cognitive abilities (Kita, 2014). Regular participation in sports exercises is associated with benefits such as emotional stability, a cheerful personality, and reduced fatigue. Individual growth cannot be separated from effective intergroup contact (Pettigrew, 1998). Human beings benefit from group integration, peer relationships, and friendships in various social, educational, and sports activities (World Health Organization, 1994; Opstoel et al., 2020). These interactions also facilitate the adjustment and selection of positive attitudes and behaviors, thereby promoting internal group identity and reducing external group bias (Pettigrew and Tropp, 2006).

In summary, sports participation, as a form of social activity, incorporates essential elements of intergroup contact. Individuals, during their engagement in sports, often encounter different groups at different times and while participating in various sports activities (such as soccer, basketball, volleyball, badminton, tennis, etc.). This gives rise to intergroup contact effects. Sports participation is also considered a positive coping mechanism, and positive interactions within sports can enhance intergroup attitudes, increase in-group identification, and reduce out-group bias, fostering positive intergroup relationships.

*Hypothesis H3a:* Sports participation among Chinese international students positively influences in-group identification.

*Hypothesis H3b:* Sports participation among Chinese international students negatively influences out-group bias.

### 2.4 In-group identification, out-group bias and cross-cultural adaptation

In-group members often bolster their psychological sense of belonging and satisfaction within their group by comparing themselves favorably to members of other social groups, providing more positive evaluations to their own group. This approach allows individuals to gain a sense of positive attachment and satisfaction, as well as obtain a certain degree of material intergroup resources (Pettigrew, 1998). De-marginalizing group identification can enhance trust between individuals and groups, leading to the attenuation of group boundaries (Li, 2022). This often requires constructing a shared group identity between individuals and group members, narrowing the gap between individuals and groups, and addressing the issue of trust deficit in intergroup relations. Toyokawa and Toyokawa (2002) indicates that among Japanese students studying in the United States, higher levels of contact with mainstream group members are associated with higher levels of life satisfaction and subjective well-being.

Intergroup contact is a crucial means of addressing out-group bias. The direct effects of intergroup contact on out-group bias indicate that limited intergroup contact hinders the formation of trust from the in-group toward the out-group, leading to an increased level of out-group bias (Schäfer et al., 2021). Lower levels of intergroup contact are associated with higher levels of out-group bias, highlighting the significant role of positive intergroup contact in alleviating intergroup relations and reducing out-group bias (Čehajić et al., 2008).

The unique requirements and paradigms of cross-cultural adaptation force international students to alter their own perceptions, reshape their behaviors in unfamiliar environments, and reconstruct cultural behaviors that tend to blend with the cross-cultural context. In summary, frequent exposure to external environments and social interactions, along with the direct or indirect acquisition of resources and experiences during this process, can enhance in-group identification, reduce out-group bias, and consequently improve international students’ cross-cultural adaptability.

*Hypothesis H4a:* In-group identification among Chinese international students positively influences cross-cultural adaptation.

*Hypothesis H4b:* Out-group bias among Chinese international students negatively influences cross-cultural adaptation.

**Figure 1:**
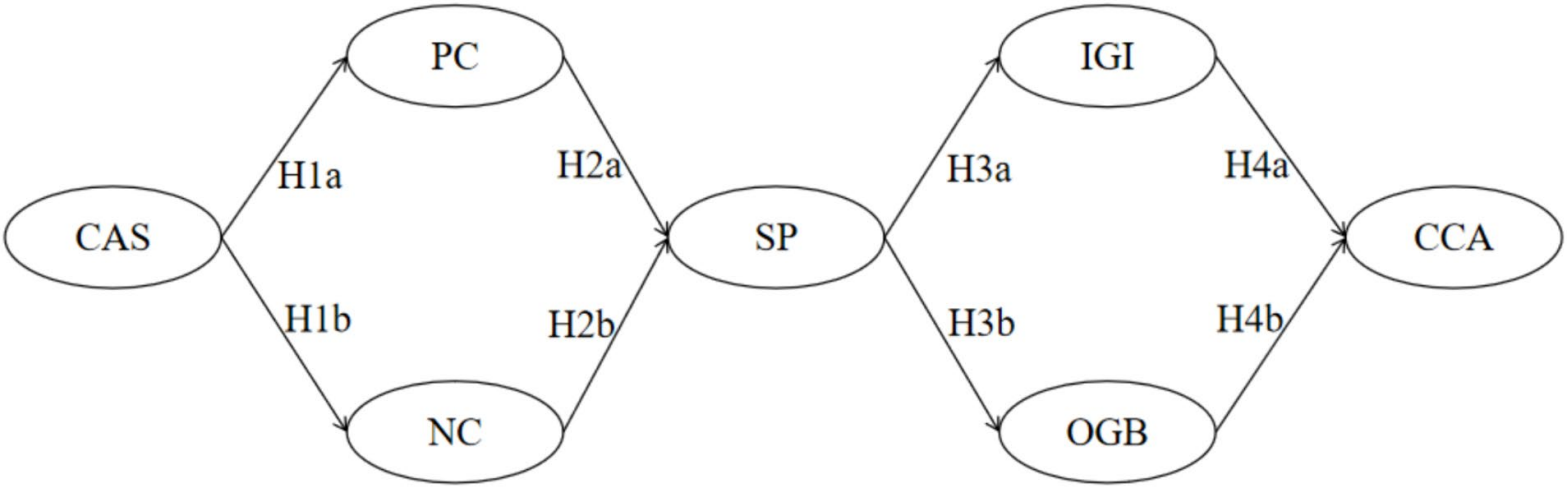
Multivariate sequential mediation hybrid model of cross-cultural adaptation for Chinese international students. CAS, Cultural Adaptation Stress; PC, Positive Coping; NC, Negative Coping; SP, Sports Participation; IGI, In-Group Identification; OGB, Out-Group Bias; CCA, Cross-Cultural Adaptation; The same applies to the following.

Integrating the above discussions, we construct a multivariate sequential mediation hybrid model for cross-cultural adaptation among Chinese international students (Figure 1). This study aims to explore the sequential mediation relationships with sports participation as the core intermediary variable. Specifically, we examine the influence of Cultural Adaptation Stress on the positive coping and negative coping strategies of Chinese International Students. Additionally, we endeavor to investigate how these coping strategies impact the intergroup relations of Chinese international students in sports participation and analyze their effects on in-group identification and out-group bias. Through this survey, we hope to elucidate the facilitating role of these factors in the cross-cultural adaptation of Chinese international students.

Sequential mediation *hypothesis H5*: Cultural Adaptation Stress has a significant impact on cross-cultural adaptation through Positive Coping, Sports Participation, and In-Group Identification.

Sequential mediation *hypothesis H6*: Cultural Adaptation Stress has a significant impact on cross-cultural adaptation through Positive Coping, Sports Participation, and Out-Group Bias.

Sequential mediation *hypothesis H7*: Cultural Adaptation Stress has a significant impact on cross-cultural adaptation through Negative Coping, Sports Participation, and In-Group Identification.

Sequential mediation *hypothesis H8*: Cultural Adaptation Stress has a significant impact on cross-cultural adaptation through Negative Coping, Sports Participation, and Out-Group Bias.

## 3 Methodology

### 3.1 Participants

This study employed convenience sampling to distribute questionnaires. Traditional random sampling methods may result in low response rates and answers that are not truthful. While theoretically all studies should use random sampling, in practice, this is nearly impossible. This is especially true for hard-to-reach populations. Convenience sampling offers advantages such as rapid implementation, low cost, and high flexibility (Guest et al., 2006). Therefore, convenience sampling was used, A total of 432 Chinese international students were selected as participants for the cross-cultural adaptation questionnaire survey, including 217 from 33 universities in South Korea, 194 from 28 universities in the United Kingdom, and 21 from 18 universities in the United States, Japan, Germany, France, Canada, Norway, Australia, and the Netherlands. The survey adopted a compensated approach, offering 5 Chinese yuan or 1,000 Korean won as remuneration. The questionnaire distribution was conducted online, with a total of 435 questionnaires distributed and collected. The number of valid questionnaires, after supplementing missing data and removing outliers, was 432, resulting in an effective rate of 99.31%.

The average age of male students is 22.99 ± 3.64 years old, with a duration of study abroad of 16.36 ± 13.89 months, and a planned duration of study abroad of 33.26 ± 19.32 months. The average age of female students is 23.00 ± 3.66 years old, with a duration of study abroad of 16.35 ± 13.95 months, and a planned duration of study abroad of 33.21 ± 19.34 months. Other information is shown in Table 1.

**Table 1:** Basic information.

	Group	Number	(%)		Group	Number	(%)
Gender	Male	183	42.40	Roommate situation	No roommate	197	45.60
	Female	249	57.60		Domestic roommate	172	39.80
Accompanied by family members	Yes	34	7.90		International roommate	63	14.60
	No	398	92.10	Employment intention	International employment	21	4.86
Current academic qualifications	Undergraduate	226	52.30		Domestic employment	333	77.08
	Master’s Student	140	32.40		Uncertain	78	18.06
	Doctoral Student	66	15.30	Adaptation status	Already adapted	327	75.70
Language proficiency level	No Degree	39	9.03		Not adapted	105	24.30
	Elementary Level 1–2	58	13.43	Five-factor model	Conscientiousness	172	39.80
	Intermediate Level 3–4	125	28.93		Openness	114	26.40
	Advanced Level 5–6	210	48.61		Extraversion	40	9.30
Accommodation status	On-campus Housing	158	36.60		Agreeableness	77	17.80
	Off-campus Renting	274	63.40		Neuroticism	29	6.70

### 3.2 Measurements

The measurement tools in this study (Table 2) consist of the cultural adaptation stress scale (Sandhu and Asrabadi, 1994), positive coping scale (Zeidner and Endler, 1995), negative coping Scale (Parker et al., 1986), sports participation scale (Sechrist et al., 1987), in-group identification Scale (Mael and Tetrick, 1992), out-group bias scale (Hewstone et al., 2002), and cross-cultural adaptation scale (Ward and Kennedy, 1999). Scoring is done using a 7-point Likert scale (1 = “completely does not apply” to 7 = “completely applies”). Regarding the reliability of the scales, we conducted Cronbach’s α reliability analysis using SPSS 23.0, and the results showed excellent reliability. This preliminary evidence suggests that the measurement tools employed in this study are suitable for investigating cross-cultural adaptation in Chinese international students.

**Table 2:** Measurement tools.

Scale name	Questionnaire items		Cronbach’ s α
Cultural adaptation stress scale	1. I have been deprived of many opportunities.	5. People show non-verbal hatred toward me.	0.96
	2. Others have prejudice against me.	6. Others do not appreciate my cultural values.	
	3. I feel that I have been treated unfairly.	7. I do not feel safe here.	
	4. I feel that what I deserve has been deprived.		
Positive coping scale	1. I take setbacks as opportunities for growth and gain experience from them.	3. I focus on taking action and strive to improve the current situation.	0.88
	2. I carefully analyze problems to better understand them.	4. I formulate plans to overcome difficulties and put them into action.	
Negative coping scale	1. I usually self-convince and endure if necessary.	3. Fantasizing about unrealistic things to alleviate distress.	0.80
	2. I silently endure the troubles in my heart.		
Sports participation scale	1. In life, I spend a lot of time engaging in physical exercise.	3. Those around me perceive me as someone who exercises regularly.	0.96
	2. I consider myself a sports and exercise enthusiast.	4. If I were forced to give up exercising, I would feel very disappointed.	
In-group identification scale	1. Through sports, in-group members will pay more attention to me.	3. Through sports, in-group members will often say ‘we’.	0.96
	2. Through sports, in-group members will praise me more.	4. Through sports, in-group members will trust me more.	
Out-group bias scale	1. Participating in sports together helps reduce discriminatory behaviors from out-group members toward me.	3. Participating in sports together helps reduce the unfair treatment from out-group members toward me.	0.97
	2. Engaging in sports together helps improve the biased attitudes of out-group members toward me.	4. Participating in sports together helps reduce the oppressive behavior from out-group members toward me.	
Cross-cultural adaptation scale	1. I have adapted to the local diet, environment, and climate.	3. I can go shopping or travel alone.	0.79
	2. I understand the requirements of the university or teachers for me.	4. I can proficiently use foreign language and make friends with local people.	

### 3.3 Data analysis

This study utilized the Windows versions of SPSS 23.0 and Amos 23.0 statistical software for analysis. Firstly, SPSS 23.0 software was employed to compute Cronbach’s α coefficient for each variable to confirm the internal consistency of the questionnaire. Descriptive statistical analysis was then conducted to understand the general characteristics of the research data. To explore the relationships between the set variables and confirm multicollinearity, correlation analysis was performed, including Cultural Adaptation Stress, Positive Coping, Negative Coping, Sports Participation, In-Group Identification, Out-Group Bias, and Cross-Cultural Adaptation. Subsequently, Amos 23.0 software was used to test the model fit and verify the sequential mediation effect, employing bootstrapping method. All input variables were standardized before analyzing to calculate standardized regression coefficients. The significance level was set at 0.05.

## 4 Results

### 4.1 Aggregated validity analysis and discriminant validity analysis

Before evaluating the structural model, it is necessary to examine the measurement model, and the selection of variables in the CFA model follows the one-stage model correction proposed by Jöreskog and Sörbom (1989). If the fit of the measurement model meets the standard, an overall structural equation model report is then conducted. Excessively high modification indices indicate correlated measurement errors between items, which may compromise the unidimensionality of the constructs. Therefore, items with excessively high modification indices and low factor loadings are deleted. The remaining results of the CFA analysis are as follows (Table 3), with factor loadings ranging from 0.57 to 0.96, composite reliabilities (CR) ranging from 0.80 to 0.97, and average variance extracted (AVE) ranging from 0.50 to 0.88. All values comply with the standards proposed by Hair et al. (1998): (1) factor loadings greater than 0.50; (2) composite reliabilities greater than 0.60; (3) average variance extracted greater than 0.50. Therefore, all seven variables in this model exhibit good reliability and convergent validity.

**Table 3:** Aggregated validity analysis.

Latent variable	Observed ariable	Unstd.	S.E.	Z	p	Std.	SMC	CR	AVE
CAS	CAS1	1.00				0.89	0.80	0.96	0.76
	CAS2	1.04	0.03	31.15	***	0.92	0.85		
	CAS3	1.08	0.03	31.29	***	0.93	0.86		
	CAS4	1.13	0.03	35.09	***	0.96	0.93		
	CAS5	0.78	0.04	19.05	***	0.73	0.53		
	CAS6	0.94	0.04	23.59	***	0.82	0.67		
	CAS7	0.97	0.04	25.21	***	0.85	0.71		
PC	PC1	1.00				0.78	0.60	0.89	0.66
	PC2	1.02	0.05	19.17	***	0.89	0.80		
	PC3	0.98	0.06	17.67	***	0.82	0.67		
	PC4	0.82	0.05	16.34	***	0.76	0.58		
NC	NC1	1.00				0.81	0.65	0.82	0.61
	NC2	1.23	0.09	14.01	***	0.92	0.84		
	NC3	0.80	0.07	11.82	***	0.57	0.33		
SP	SP1	1.00				0.93	0.86	0.96	0.85
	SP2	1.05	0.03	37.66	***	0.95	0.89		
	SP3	1.06	0.03	36.43	***	0.94	0.88		
	SP4	0.99	0.03	30.24	***	0.88	0.78		
IGI	IGI1	1.00				0.90	0.81	0.96	0.84
	IGI2	1.04	0.03	32.93	***	0.94	0.88		
	IGI3	1.01	0.03	29.55	***	0.90	0.81		
	IGI4	1.03	0.03	31.69	***	0.93	0.86		
OGB	OGB1	1.00				0.94	0.89	0.97	0.88
	OGB2	0.99	0.03	37.32	***	0.93	0.86		
	OGB3	0.99	0.03	39.14	***	0.94	0.88		
	OGB4	0.97	0.03	37.64	***	0.93	0.87		
CCA	CCA1	1.00				0.79	0.62	0.80	0.50
	CCA2	0.98	0.08	12.83	***	0.70	0.49		
	CCA3	0.92	0.08	11.45	***	0.61	0.38		
	CCA4	0.87	0.07	13.22	***	0.73	0.53		

***p<0.001.

As shown in Table 4, the absolute values of skewness range from 0.02 to 1.45, which are less than 2.00, and the absolute values of kurtosis range from 0.74 to 2.01, which are less than 8.00. Therefore, this data can be considered as normally distributed (Zhang and Ronghai, 2020). The scores for the seven latent variables range from 3.93 to 5.92, indicating that all seven latent variables, from cultural adaptation stress to cross-cultural adaptation, fall within a positive evaluation range. Additionally, discriminant validity analysis was conducted using the AVE method. For each variable, the square root of AVE should be greater than the correlation coefficients with each pair of variables, indicating discriminant validity among the variables. In Table 4, the diagonal values, representing the square root of AVE for each variable, are greater than the standardized correlation coefficients with other variables. The correlation coefficients are all below 0.70, demonstrating discriminant validity among the variables and the absence of multicollinearity. This further underscores the reliability of the model.

**Table 4:** Discriminant validity analysis.

	M	SD	Skewness	Kurtosis	AVE	CAS	PC	NC	SP	IGI	QGB	CCA
CAS	3.08	2.08	0.70	−0.83	0.76	**0.87**						
PC	5.36	1.71	−1.20	0.74	0.66	−0.20**	**0.81**					
NC	4.48	2.05	−0.37	−1.03	0.61	0.21**	0.22**	**0.78**				
SP	3.93	2.23	0.02	−1.47	0.85	−0.06	0.20**	0.03	**0.92**			
IGI	5.03	1.92	−0.78	−1.99	0.84	−0.10*	0.30**	0.19**	0.32**	**0.92**		
QGB	4.62	2.06	−0.46	−1.09	0.88	−0.07	0.26**	0.18**	0.19**	0.60**	**0.94**	
CCA	5.92	1.34	−1.45	2.01	0.50	0.01	0.33**	0.24**	0.02	0.30**	0.21**	**0.71**

**p<0.01, *p<0.05. The bold italic represents the square root values of Average Variance Extracted (AVE).

### 4.2 Research hypotheses H1a ~ H4b analysis

This study employed the Harman’s single-factor test to examine common method bias. The results indicate that the first factor, without rotation, explains only 25.49% of the total variance, which is less than 40%. This suggests the absence of a significant common method bias in this study. With a sample size of 432 and seven latent variables, the potential risk of poor model fit due to the large sample size and numerous variables is acknowledged, potentially leading to an inflated *χ^2^*. However, the model fit assessed by AMOS 23.0 meets the standard criteria, as demonstrated in the results (Table 5), confirming the excellent fit of the model.

**Table 5:** Fitted value.

*χ* ^2^	df	*χ*^2^/df	GFI	AGFI	CFI	NFI	TLI	RMSEA
1092.24	397	2.75	0.86	0.83	0.94	0.91	0.94	0.06

Among them, the prediction (γ) of cultural adaptation stress on positive coping (H1a) is −0.20, *p* < 0.001; the prediction (γ) of cultural adaptation stress on negative coping (H1b) is 0.19, *p* < 0.001; the prediction (γ) of positive coping on sports participation (H2a) is 0.26, *p* < 0.001; the prediction (γ) of negative coping on sports participation (H2b) is −0.05, *p* < 0.05; the prediction (γ) of sports participation on in-group identification (H3a) is 0.34, *p* < 0.001; the prediction (γ) of sports participation on out-group bias (H3b) is 0.20, *p* < 0.05; the prediction (γ) of in-group identification on cross-cultural adaptation (H4a) is 0.31, p < 0.001; and the prediction (γ) of out-group bias on cross-cultural adaptation (H4b) is 0.05, *p* > 0.05. In summary, hypotheses H1a, H1b, H2a, H2b, H3a, H3b, and H4a are supported, while hypothesis H4b is not supported.

#### 4.2.1 Explanation of H1a and H1b

According to the stress coping theory, individual cognitive differences influence their coping tendencies when facing stress, whether inclined toward positive coping or leaning toward negative coping (Anderson, 2004). The results of this study indicate that both H1a and H1b are statistically significant (*p* < 0.001). However, the predicted (γ) value for H1a is −0.20, suggesting a negative correlation between cultural adaptation stress and positive coping among Chinese international students. On the other hand, the predicted (γ) value for H1b is 0.19, indicating a positive correlation between cultural adaptation stress and negative coping. In other words, when stress is moderate, Chinese international students are inclined to adopt positive coping strategies; however, when stress becomes overwhelming, they tend to resort to negative coping mechanisms. This finding aligns with previous research (Bahrke and Morgan, 1978).

#### 4.2.2 Explanation of H2a and H2b

The predicted (γ) value for H2a is 0.26, indicating that positive coping promotes sports participation, while the predicted (γ) value for H2b is −0.05, suggesting that negative coping hinders sports participation. H2a (*p* < 0.001) is significantly more pronounced than H2b (*p* < 0.05), indicating that Chinese international students are more inclined to adopt positive coping strategies. The predicted (γ) value for H2a is much greater than the predicted (γ) value for H2b, suggesting that students who employ positive coping mechanisms such as seeking help and problem-solving are more likely to engage in physical exercise compared to those who use avoidance and fantasy, representing a negative coping approach. This finding is consistent with prior research (Printz et al., 1999). Under stress, Chinese international students who adopt positive coping strategies demonstrate a problem-solving orientation, promoting psychological development and positive intergroup interactions. In other words, in a positive state characterized by vitality, enthusiasm, and optimism, individuals are more actively involved in external physical activities. Conversely, in a negative state characterized by tension, anxiety, and depression, participation in sports is often rejected.

#### 4.2.3 Explanation for H3a and H3b

According to the Intergroup Contact Theory, intergroup contact and social identification are crucial for fostering intergroup trust and reducing intergroup bias (Amir, 1976). The predicted (γ) coefficients for both H3a and H3b in this study are positive, indicating that, in the context of sports participation, under appropriate conditions, individual contact with the in-group can effectively increase intergroup trust, reduce bias and intergroup tension, and improve attitudes toward out-groups (Stephan and Stephan, 1985). Furthermore, the predicted (γ) value for H3a is greater than the predicted (γ) value for H3b. H3a (*p* < 0.001) is significantly more pronounced than H3b (*p* < 0.05), revealing that the explanatory power and predictability of sports participation on in-group identification far exceed that on out-group bias. Chinese international students are more inclined to engage in sports interactions with their fellow countrymen. Therefore, governments and schools should actively promote and organize sports-related activities among students from different countries, both on and off-campus, encourage sports interactions with local residents, and thereby reduce out-group bias.

#### 4.2.4 Explanation for H4a and H4b

The predicted (γ) value for In-Group Identification on Cross-Cultural Adaptation (H4a) is 0.31, *p* < 0.001, while the predicted (γ) value for Out-Group Bias on Cross-Cultural Adaptation (H4b) is 0.05, *p* > 0.05. The results indicate that H4a has the highest explanatory power among all predicted (γ) values in this model, while H4b (*p* > 0.05) suggests that out-group bias does not have a significant impact on cross-cultural adaptation. Combining the earlier statement that “Chinese international students are more inclined to interact with fellow countrymen” with the previous information about the duration of study abroad (SD = 16.4 months) and roommate situations (only 14.6% have foreign roommates), it suggests that Chinese international students, over an extended period, tend to derive intergroup resources from their own in-group. The cross-cultural adaptation of Chinese international students is dependent on their in-group, and a well-established social network with out-group members has not yet been formed. This makes out-group bias unable to influence cross-cultural adaptation. Therefore, there is an urgent need to establish a reasonable mechanism for intergroup communication.

### 4.3 Sequential mediation effects analysis

To more accurately calculate the mediation effects, the Bootstrap estimation method was first used to estimate the standard errors of the mediation effects, followed by further calculating the significance level of the mediation effects. The results (Table 6) indicate that the total effect of cultural adaptation stress on cross-cultural adaptation is −0.004, with a standard error of 0.002. The absolute value of the Z score is 2.000, meeting the standard of being greater than 1.96. At a 95% confidence interval level, the lower limit of the confidence interval obtained by the Bias-corrected estimation method is −0.009, the upper limit is −0.001, excluding 0. Moreover, the total effect is significant with *p* < 0.01, confirming the validity of the overall model effect.

**Table 6:** Sequential mediation effects analysis.

Sequential mediation visualization diagram		PE	S.E.	*Z*	Bias-corrected 95% CI		*p*	Validity
					Lower	Upper		
H5	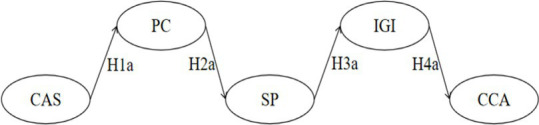	−0.003	0.002	−1.500	−0.008	−0.001	0.000^***^	Yes
H6	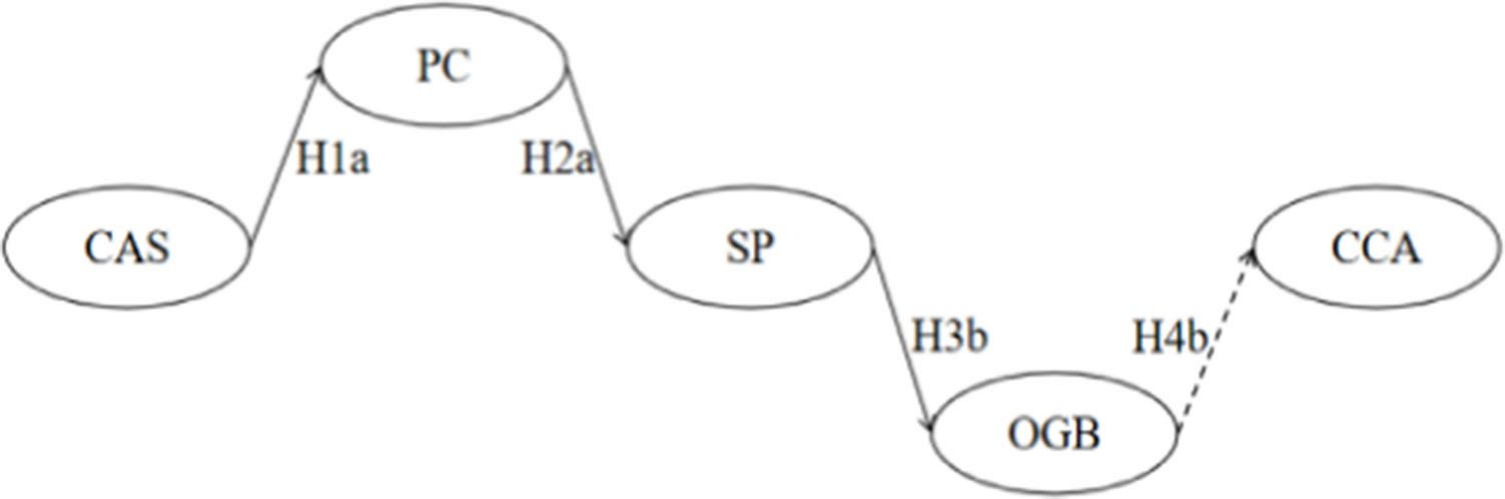	0.000	0.000	0.000	−0.002	0.000	0.209	No
H7	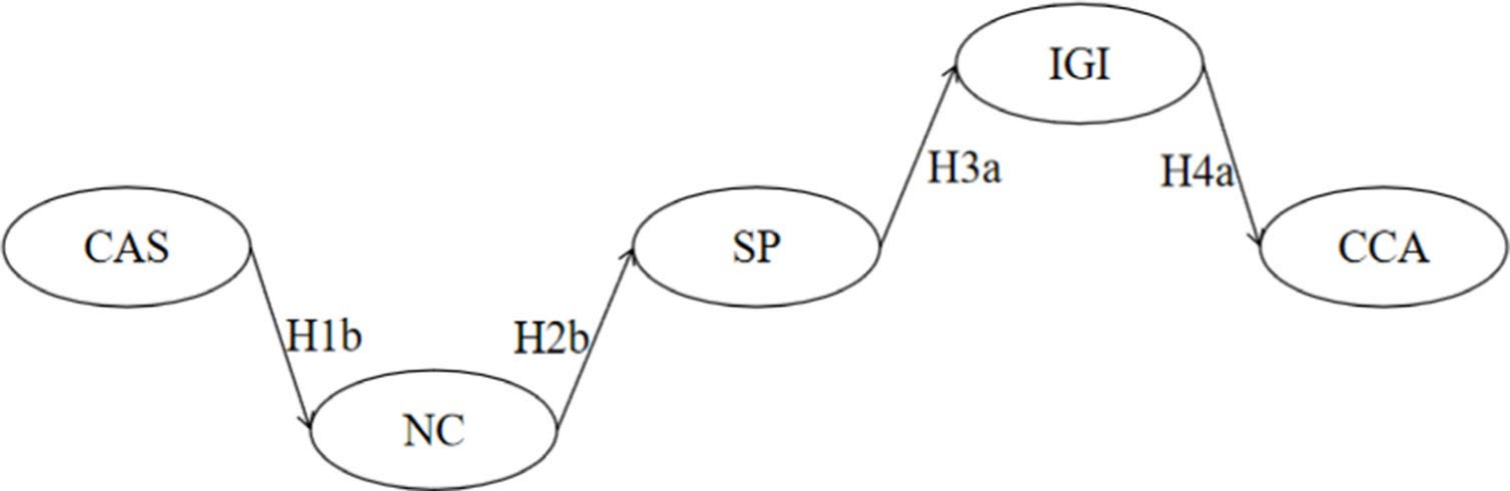	0.000	0.001	0.000	−0.002	−0.001	0.027^*^	Yes
H8	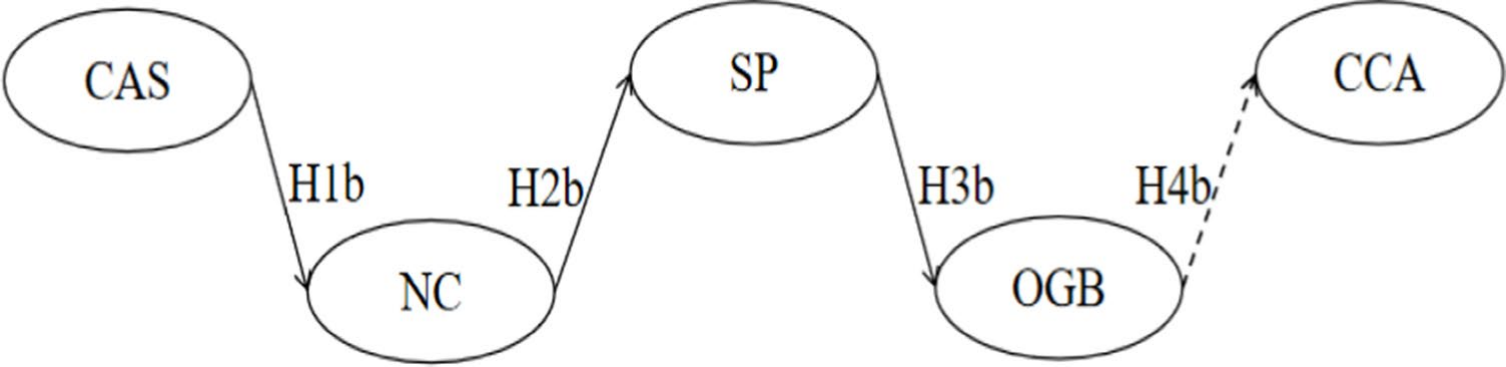	0.000	0.000	0.000	−0.001	0.000	0.244	No
Total		−0.004	0.002	−2.000	−0.009	−0.001	0.002^**^	Yes

Dashed line *p*>0.05, ***p<0.001, **p<0.01, *p<0.05.

**Figure 2:**
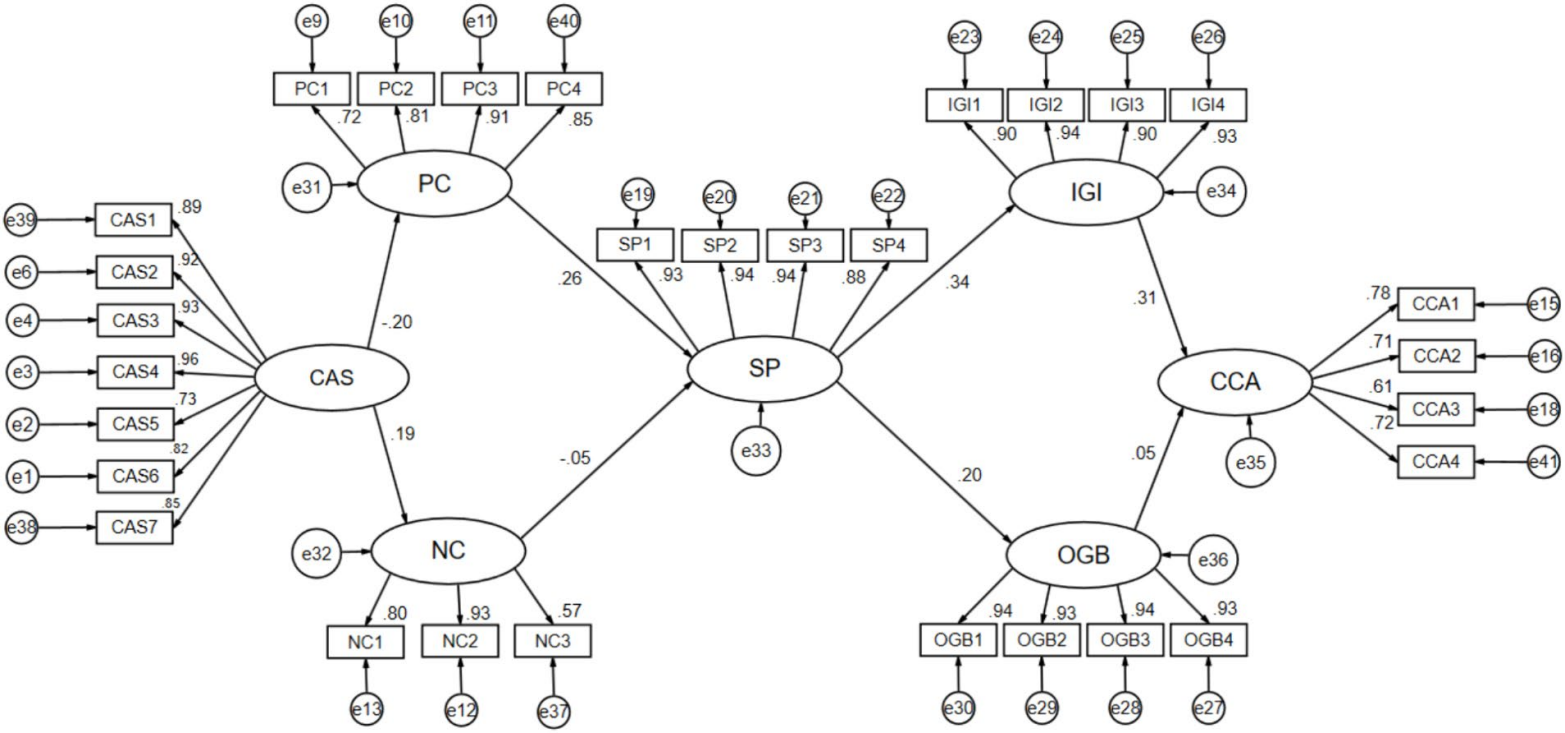
Multivariate sequential mediation mixed-effects model diagram for cross-Cultural adaptation among Chinese international students.

Taking an overview of the entire model (Figure 2) and (Table 6), it can be observed that cultural adaptation stress is associated with positive coping (H1a), negative coping (H1b), positive coping (H1a), negative coping (H1b), and sports participation (H2a). Additionally, sports participation is linked with in-group identification (H3a), and in-group identification is connected to cross-cultural adaptation (H4a). Each of these stages, whether individually or in a chained sequence, demonstrates statistical significance, providing dual confirmation for the establishment of the sequential mediation effects in both H1a-H2a-H3a-H4a and H1a-H2b-H3a-H4a.

However, the results of the research hypotheses indicate that out-group bias and cross-cultural adaptation (H4b) lack significance, suggesting that external group bias has no impact on the cultural adaptation of Chinese international students. Sequential mediation effects results show that the sequential mediation effects in H1a-H2a-H3a-H4a and H1a-H2b-H3a-H4a are established, while the other two sequential mediation effects starting from Cultural Adaptation Stress (CAS), passing through Positive Coping (PC), Negative Coping (NC), and Sports Participation (SP), are not established. They are interrupted at the connection point between Out-Group Bias (OGB) and Cross-Cultural Adaptation (CCA). Therefore, efforts should be made to overcome the dilemma that “out-group bias cannot effectively predict cross-cultural adaptation” and strive for a sustained transformation between in-group and out-group.

## 5 Discussion

The analysis of sequential mediation effects on cross-cultural adaptation reveals the following findings: When cultural adaptation stress is moderate, adopting positive coping strategies enhances sports participation. Conversely, when cultural adaptation stress is excessive, adopting negative coping strategies hinders sports involvement. Sports participation contributes to increased internal group identity and reduced external group bias. Internal group identity further promotes cross-cultural adaptation, while predicting cross-cultural adaptation based on external group bias proves challenging.

The individual’s cognitive differences influence their coping tendencies during stress, leading either toward positive or negative coping strategies. The results of this study indicate a negative correlation between cultural adaptation stress and positive coping among Chinese international students. Conversely, there is a positive correlation between cultural adaptation stress and negative coping. In other words, when the stress is moderate, Chinese international students tend to adopt positive coping strategies, while in situations of excessive stress, they tend to resort to negative coping mechanisms. Positive coping strategies facilitate sports participation, while negative coping hinders it (Hayes et al., 2015; Moen et al., 2019). When cultural adaptation stress occurs, Chinese international students who employ positive coping mechanisms are more likely to engage in sports activities compared to those who use avoidance or fantasy, which are considered negative coping strategies. Through sports participation, there is an effective increase in internal group identity, a decrease in external group bias, and a promotion of cross-cultural adaptation (Pettigrew and Tropp, 2006).

However, external group bias cannot impact cross-cultural adaptation. This study reveals that Chinese international students primarily acquire social resources from their relevant internal groups. The cross-cultural adaptation of Chinese international students depends on internal group dynamics, and external group bias fails to explain their cross-cultural adaptation. This finding is consistent with past research results, such as Chinese international students in New Zealand seeking educational support, social support, and emotional support from fellow internal group members through SNS (Cao and Zhang, 2012). Firstly, previous studies predominantly focused on the populations of their own countries and regions rather than specifically targeting international students in cross-cultural research. Secondly, due to language barriers, international students are unable to interact with local people in the short term. Additionally, their living environment encourages communication in their native language, with dormitory roommates or shared apartment mates often being fellow nationals. These multiple factors result in the social circles of international students being more oriented toward interaction and communication with fellow nationals and local classmates. Thirdly, the cultural similarities and linguistic commonality among fellow nationals foster a sense of internal group identity. Lastly, from the perspective of shared characteristics, common political systems, cultural connotations, and physical features among fellow nationals may contribute to a subconscious tendency to exclude external groups.

Between cultural adaptation pressure and cross-cultural adaptation, positive coping, sports participation, and internal group identity have a significantly positive mediating effect. Therefore, one can accelerate the cross-cultural adaptation of Chinese international students through the following aspects.

Firstly, it is crucial to address the problems and challenges encountered by international students in the process of cross-cultural adaptation. This can be achieved by developing mechanisms for cultural exchange between the home and host cultures and establishing communication platforms. To better overcome the barriers of cross-cultural interactions, a targeted understanding of the root causes of issues is needed. Developing feasible communication mechanisms and establishing a diversified international cross-cultural communication system, including real-time communication, language translation, and geographical positioning, will help address various challenges that international students may face before, during, and after their studies. Using technological means to protect the rights of international students can contribute to resolving their difficulties.

Secondly, participating in sports activities enables Chinese international students to better integrate into the local culture and enhance interactions with local people. Sports activities serve as an opportunity to promote cultural identification between internal and external groups, laying the foundation for cross-cultural adaptation. This implies that, in appropriate communication environments, students can use sports activities to share information with local individuals, facilitating cultural exchange and integration. This, in turn, allows them to better adapt to the new cultural environment and ensures stability and safety throughout their cross-cultural experience.

Lastly, expanding the scope of identification between international students and internal/external groups is essential. Research results indicate that Chinese international students find it easier to reach consensus with their compatriots, while interactions with local individuals are limited. To address this issue, it is necessary to establish more communication channels, showcase the unique aspects of Chinese culture, and introduce both the differences and similarities with foreign cultures. By fostering a better understanding and integration between internal and external groups, a smoother and more enduring cross-cultural group communication can be achieved, ensuring the adaptation and continuity of international students in the new cultural context.

## 6 Future prospects and research limitations

In the relationship between cultural adaptation stress and cross-cultural adaptation, there are still many influencing factors to be explored, among which personality traits and duration of study abroad are hot topics in the research on cultural adaptation. Although the categorical variables of personality traits and duration of study abroad for Chinese international students were investigated in this study, their relationship with the cross-cultural adaptation of Chinese international students has not been studied yet. Given that the majority of participants are from South Korea and the United Kingdom, concerns arise about potential biases in the study’s findings due to the uneven distribution across countries. Efforts will be made to achieve a more balanced representation across nations to ensure the generalizability of results.

This paper has some limitations and suggestions for future research. Firstly, the study only explores sequential-mediated effects, and subsequent research will test and analyze the direct effects between explanatory variables and dependent variables. Secondly, there has not been in-depth research on how Chinese international students, when facing cultural adaptation stress, adopt different coping strategies based on their personality traits. Thirdly, as the duration of study abroad for Chinese international students continues to increase, there is a need for research on the stages and nodes of changes in cross-cultural adaptation. Fourthly, whether the multivariate sequential-mediated mixed model for cross-cultural adaptation of Chinese international students is applicable to the entire international student population is unknown. Its universality requires further discussion, and it is hoped that future research will collect data from the entire international student population for validation and discussion. Lastly, a longitudinal study that spans from the past to the future should be conducted to form a series of cross-cultural adaptation processes. This will better guide the cross-cultural adaptation of Chinese international students and, in turn, promote the dissemination of Chinese cultural concepts through international students as cultural carriers.

## Data availability statement

The raw data supporting the conclusions of this article will be made available by the authors, without undue reservation.

## Ethics statement

Ethical review and approval was not required for the study on human participants in accordance with the local legislation and institutional requirements. Written informed consent from the patients/participants or patients/participants’ legal guardian/next of kin was not required to participate in this study in accordance with the national legislation and the institutional requirements.

## Author contributions

CM: Conceptualization, Data curation, Formal analysis, Investigation, Methodology, Software, Validation, Visualization, Writing – original draft, Writing – review & editing. SZ: Conceptualization, Data curation, Methodology, Supervision, Validation, Visualization, Writing – review & editing.
